# Beethoven’s death—the result of medical malpractice?

**DOI:** 10.1007/s10354-021-00833-x

**Published:** 2021-03-25

**Authors:** Christian Reiter, Thomas Prohaska

**Affiliations:** 1 Center for Forensic Medicine, Medical University of Vienna Center for Forensic Medicine, Medical University of Vienna, Sensengasse 2, 1090 Vienna, Austria; 2 Chair of General and Analytical Chemistry, Montanuniversität Leoben, 8700 Leoben, Austria

**Keywords:** Ludwig van Beethoven, Hair analysis, LA-ICP-MS, SEM, Iatrogenic lead intoxication, Ludwig van Beethoven, Haaranalyse, LA-ICP-MS, REM, Iatrogene Bleivergiftung

## Abstract

Two different strands of hair taken from Beethoven’s head after his death were examined for heavy metals using scanning electron microscopy (SEM) and laser ablation-ICP-MS (inductively coupled plasma–mass spectroscopy). The results revealed the presence of small lead particles on the surface of Beethoven’s hairs and fluctuating lead levels in hair medulla along the length of the hair due to alternating lead exposure, with an average lead exposure of 100 times the normal value. The time-line attached to the peaks of these fluctuating values correlate with the pneumonia treatment and the paracenteses performed, including the subsequent treatment of the procedure wounds. While the administration of lead-containing drugs and treatments had been proven to resolve the pneumonia, it had simultaneously caused massive liver failure, accelerated by pre-existing cirrhosis. The question as to whether Beethoven’s death was a case of malpractice can only be answered from a forensic point of view ex ante, since the state of the medical knowledge of the time has to be taken into account.

## Abstract (de)

Zwei verschiedene Haarsträhnen, die postmortal vom Kopf Beethovens entnommen worden waren, wurden mittels REM (Rasterelektronenmikroskop) und Laserablation-ICP-MS („inductively coupled plasma–mass spectroscopy“) auf Schwermetalle untersucht. Die Ergebnisse zeigten das Vorhandensein kleinster Bleipartikel auf der Oberfläche der Haare und eine Bleibelastung des Haarmarks aufgrund fluktuierender Bleiexposition, wobei die durchschnittliche Bleiexposition das 100-fache des Normalwerts betrug. Der zeitliche Verlauf der Spitzenwerte dieser ondulierenden Messergebnisse korreliert mit der Behandlung der Lungenentzündung und den durchgeführten Parazentesen sowie den anschließenden Wundbehandlungen. Während die Anwendung bleihaltiger Medikamente und Behandlungen die Pneumonie nachweislich zum Abklingen brachte, bewirkte sie gleichzeitig ein schweres Leberversagen, das durch die bereits vorbestehende Zirrhose beschleunigt wurde. Die Frage, ob Beethovens Tod die Folge ärztlichen Fehlverhaltens war, kann aus forensischer Sicht nur ex ante beantwortet werden, da der Stand des medizinischen Wissens der damaligen Zeit zu berücksichtigen ist.

## Introduction

When discussing the life and works of Ludwig van Beethoven, one cannot ignore the bewildering details of his biography concerning the contrast between his deteriorating hearing on the one hand and the excelling mastery of the musical arts on the other. The most up-to-date position regarding Beethoven’s etiology of illness dates back to 1987 as reviewed in the monograph *Die Krankheiten Ludwig van Beethovens* written by Bankl and Jesserer [1]. Regardless of the conclusions published in this work, speculations and conjectures concerning Beethoven’s medical history have continued to surface. Uncontested throughout, however, was the cause of the scars on his face from the case of pox Beethoven had suffered from as a child, and the constant consumption of alcohol, which was a habit obviously adopted from his father. At untimely age of 16 (in 1786) it was noticed that Beethoven had been showing signs of hearing impairment during public encounters. Since 1795, aged 25, Beethoven had been suffering from recurring digestion problems, accompanied with abdominal cramps.

Aged 26 it was clear, at least from Beethoven’s point of view, that he was starting to show symptoms of going deaf. In 1802 at the age of 32, Beethoven had conveyed his condition in the famous “Heiligenstädter Testament”, an unsent letter he wrote to his brothers Carl and Johann. In this presuicidal letter, Beethoven had expressed his desire to continue his artistic legacy despite his hear loss.

Starting in 1813, aged 43, Beethoven had been using different contraptions, some more successful than others, in hope of finding aid to his hearing problems.

In 1818, aged 48, Beethoven had become completely deaf, and from this moment on he used his “conversation books” through which he communicated with people around him. Although many are missing, the remaining ones provide a vivid glance into his experience in the last years of his life. In an entry dated back to 1821, a “rheumatic attack” was reported, which was mainly characterized by the occurrence of jaundice and indigestion.

In 1825, occurrence of nosebleeds, vomiting of blood and diarrhea were recorded.

In February 1826, episodes of diarrhea, spasms in the hands and feet, vertigo, back pain and a presence of a bitter aftertaste had also appeared. At the end of September in that same year, Beethoven had retreated to his brother Johann in Gneixendorf, north of Krems (Lower Austria), where he had continued to suffer from various symptoms, including swelling in his legs.

Although experiencing a lack of appetite accompanied with diarrhea, his abdominal circumference, however, had seemed to increase. Throughout this period, his habit of drinking large amounts of wine had remained unchanged.

In the winter of 1826/27, Beethoven decided to relocate to Vienna and had himself transported with a wagon. At that time, one was required to make an overnight stop when traveling this route. Due to the use of an open carriage and bad weather conditions, the soaking wet Beethoven was forced to take shelter in an unheated local inn.

The result of this stay was pneumonia, which was diagnosed on December 2nd. However, the search for medical assistance had taken longer than usual because Beethoven’s difficult character had deemed him an unwanted patient for many doctors. For this reason, it had taken a few days until Dr. Wawruch, a professor working as an internist in the surgical clinic at the Garnisonsspital, had agreed to take him on as a patient.

In a piece of writing [2] published after Wawruch’s passing dated 20 May 1827 Wawruch mentioned that he had prescribed Beethoven a “rigorous antiphlogistic therapy”. This treatment had subsequently brought upon what had seemed to be an improvement in the following days (10th to 12th of December). However, this relief had been deemed to be temporary, as Beethoven’s condition worsened. He had begun to suffer from jaundice, diarrhea and vomiting as well as reaching unprecedented levels of urinary retention and ascites. In light of his lung infection and severe ascites, his physician chose to seek consult with the surgeon Dr. Seibert, who had performed a series of paracenteses on Beethoven beginning on 20 December 1826. Through this procedure, the fluid building up in Beethoven’s abdominal cavity was relieved. However, this procedure has left him with a local bacterial infection, erysipelas, which caused skin inflammation around the puncture site.

Around the same time, a friend of Beethoven, Johann Nepomuk Hummel, and his 15-year-old pupil Ferdinand Hiller had travelled to Vienna. The two had made visits to Beethoven on the 8th, 13th and 23rd of March 1827. On the 26th of March 1827, Beethoven had passed away.

In his “Heiligenstädter Testament” written in 1802, Beethoven had expressed his last wish in writing, to allow posterity, with the aid of science, to find the cause of his deafness. Following his plea, an autopsy was performed on 27 March 1827 by Josef Wagner, assistant at the Vienna General Hospital’s Museum for Pathology. The document of this autopsy is still kept at the Federal Pathologic–Anatomical Museum (Narrenturm) in Austria. The result of this postmortem examination revealed signs of a nodose cirrhosis of the liver, combined with severe ascites, fibrosis and chronic inflammation of the pancreas, enlargement of the spleen, renal pelvis calculi, and atrophic acoustic nerves on both sides, with enlarged blood vessels of both petrosal bones.

## Material

### Guevara’s strand of hair

On 27 March 1827 Ferdinand Hiller was permitted to remove a lock of Beethoven’s hair for safekeeping. Ferdinand von Hiller (1811–1885), a member of the Mosaic creed, became the head of a family of musicians; he bequeathed the locket containing Beethoven’s hair in the form of a birthday present to his son, Paul Hiller, on the 1 May 1883 [3]. Subsequently the records of the Hiller family in Germany were lost in the years before WWII. However, records resurfaced showing that since October 1943 the locket was in the possession of the physician Kay Alexander Fremming, at the north coast of Denmark. At that time, Dr. Fremming had a significant role in helping Jews escape from the Nazi regime to Sweden. The locket was put up for auction on behalf of the Fremming family at Sotheby’s London auction house. It was purchased on 1 December 1994, by members of the American Beethoven Society for the bidding price of 3600 British pounds. The main financiers of this bid were the real estate brokers Ira F. Brilliant and the urologist Alfredo Guevara. On 12 December 1995, 160 of 582 hairs were extracted from the locket at the University of Arizona and handed over to the private ownership of Alfredo Guevara. The remaining 422 hairs, locket and authenticity certificate included (found inside the locket) were entrusted to the Ira F. Brilliant Center of Beethoven studies at the San Josè University Museum. As a man of medicine and science, Alfredo Guevara felt obligated to pursue Beethoven’s request in conducting a scientific investigation in order to find the source of his illness.

In 2000, some of the hairs owned by Guevara were ran though the particle accelerator at the Argonne National Laboratory. This sample proved a lead content in the hair which is a hundred times higher than a normal reading (60 ppm compared to 0.6 ppm). Due to the fact that cases of hearing loss associated with lead poisoning were previously mentioned in the medical literature [4], the findings of this test fuelled the hypothesis that Beethoven’s deafness might have been related to lead poisoning.

The body of Ludwig van Beethoven was buried on 29 March 1827 at the Währinger Cemetery; now Schubert Park in the 18th district of Vienna.

The first exhumation followed by reburial had taken place on 13 October 1863. At the exhumation, Dr. Romeo Seligmann, later a professor for the history of medicine, was present. In the course of this exhumation, several fragments of Beethoven’s skull came into his possession [5]. These fragments remain till this day in the possession of his descendants, who recently dedicated them to the Ira F. Brilliant Center of Beethoven Studies. The pathologist Hans Bankl could confirm that the fragments in Seligmann’s possession were in fact those originating from Beethoven’s skull, due to the fact that a plaster cast of the original skull, which is located in the Federal Pathologic–Anatomical Museum in Vienna, were missing the same skull fractions, which had been replaced with moulding compound by the moulageur. According to a DNA test later conducted on the Seligmann fragments, there is a molecular biological match when compared to the hairs owned by Guevara.

In 2004 the story of the aforementioned locket containing Beethoven’s hairs was presented in a Canadian TV film [6], whose production team came to Vienna to investigate the possible causes that were the source of Beethoven’s exposure to high levels of lead throughout his life. Since at that time, the first author had already dealt with some criminal cases together with the University of Natural Resources and Life Sciences, after the presence of heavy metal poisoning [7], the TV crew was referred to him.

Due to the fact that the hair ran through the particle accelerator was examined as a whole, the results could not provide an explanation regarding any acute or chronic lead poisoning. In order to investigate the chronology of the possible lead poisoning prior to his death, an additional analysis which examines the hair along different sections of its growth was necessary. Since it is known that during its growth process, the level of active ingredients that are contained in a hair is an indication of their concentrations in the body, which are absorbed from the hair root itself, and, thus, provide us with an appropriate time scale of the substance’s presence in the body by separately comparing and analyzing different hair sections at a time [8]. We subsequently received 2 hairs (42 mm and 98 mm long without roots) from Dr. Guevara in order to conduct an analysis on condition that they be returned post examination.

## Rollett Museum’s strand of hair

In the Beethoven memorial at the former estate Erdödy in Alt Jedlesee (21st district of Vienna), where Beethoven was a frequent guest, a lock of Beethoven’s hair that was released by the Rollett Museum in Baden is on display. The lock of hair was cut from Beethoven’s head on 27 March 1827 by Josef Karl Bernard the editor-in-chief of the *Wiener Zeitung*. His granddaughter Marie Emerice de Bersuder gave it to the mother of her French pupil Katharina Hansy-Odorfer in Baden in 1923. In 1947, Mrs. Hansy-Odorfer donated the lock of hair to the municipality of Baden (Rollett Museum). In contrast to Guevara’s samples, these hairs are up to 155 mm long, but similarly without roots.

## Methods

**Fig. 1:**
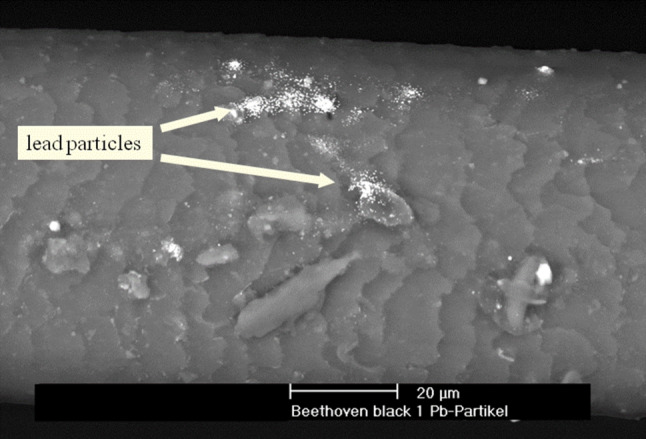
Numerous tiny lead particle deposits on the surface of Beethoven’s hairs. SEM

A superficial examination of the hairs by means of a scanning electron microscope (SEM) without prior opacification in combination with energy-dispersive X‑ray analysis (SEM-EDS) done at the University of Applied Arts, Institute for Technical Chemistry by Prof. A. Vendl revealed numerous round lead particle deposits ranging in size of 0.1 to 2 microns on the surface of the hairs (Fig. 1; [9]).

**Fig. 2:**
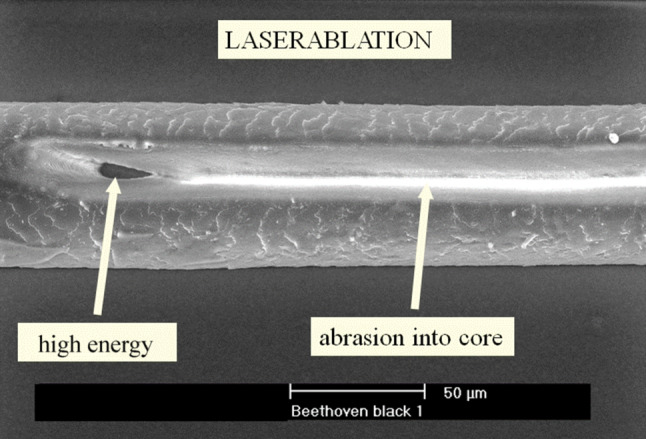
The appearance of laser ablation on Beethoven’s hair as a function of energy used. SEM

After a chemical rinse of the hairs to rid it of the superficial lead impurities, an analysis by means of laser ablation-ICP-MS (inductively coupled plasma–mass spectroscopy) was performed by the second author at the University of Natural Resources and Life Sciences, Analytical Chemistry. In this examination technique [8], the evaporation of the hair matrix is conducted with the aid of a high-energy laser beam and an optical instrument placed under the microscope in order to produce the desired effect, wherein the evaporation residues of this procedure are further analyzed by ICP-MS. The diameter of the laser beam, depending on the setting, is about 20 microns, so that, depending on the applied energy, grooved ablations of hair cortex or medulla of hair, as well as needle eye like “slugs” can be created along the hair (Fig. 2).

In this manner, it is possible to make up to 40 single measurements for each millimeter of hair in the longitudinal direction without causing discontinuity and thereby, depending on the energy used, make separate analytical conclusions about the chemical nature of the hair cortex, as well as the hair medulla.

## Results

The hair provided by Dr. Guevara were around 42 and 98 mm long and were supposed to have been cut off by Hiller directly at the level of Beethoven’s scalp. The interpretation of analytical results throughout the subsections has shown that Beethoven’s hair had lead concentrations that were elevated by a factor of 100. The lead concentration of the hairs, however, was not increased uniformly over the entire length. Human hair follicles are embedded in the skin approximately 3–4 mm below the surface. If a hair sample has been cut off at the skin level, about 3–4 mm must be added to obtain the total length. The range of hair growth rate reported for scalp hair is somewhat dependent on ethnicity, location on head, sex, season and age. In Caucasian men an average growth rate of scalp hair of 0.37 mm (range 0.16–0.50 mm) per day was found [10].

**Fig. 3:**
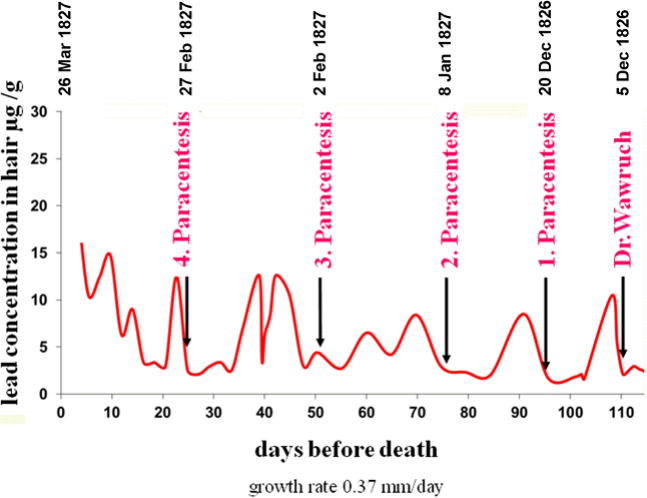
Time-dependent lead loading of the hair “Guevara 1” combined with the dates of medical measures taken

Taking into account the actual and corrected length of the samples as well as slightly variable growth rates depending on the extraction sites of head, there are 9 to 10 peak loads in the estimated 110–120 days before Beethoven’s death. It is possible to correlate these lead peaks to the course of medical treatment reconstructed from the descriptions of the course of treatment in the conversation books (Fig. 3). At an average growth rate of human scalp hair, the first significant lead exposure appears to occur about 110 days before Beethoven’s death. This would correlate to about 5 December 1826, which was around the time that Dr. Wawruch had administrated the “anti-inflammatory therapies” to treat Beethoven’s pneumonia. The next peak in lead exposure occurred around 20 December 1826 after Dr. Seibert performed the first paracentesis. The second paracentesis was performed on 8 January 1827 and, immediately thereafter, around the 70th and 60th day before Beethoven’s death, there are further significant events of lead exposure. The third paracentesis was performed on 2 February 1827, which was about 50 days before Beethoven’s death, whereupon around the 45th and 38th day we can observe two more massive lead peaks in the hair analysis. After the fourth paracentesis, on 27 February, 1827, a massive lead exposure took place again, which then continued from day 15 before Beethoven’s death and persisted with a steady increase until his death.

**Fig. 4:**
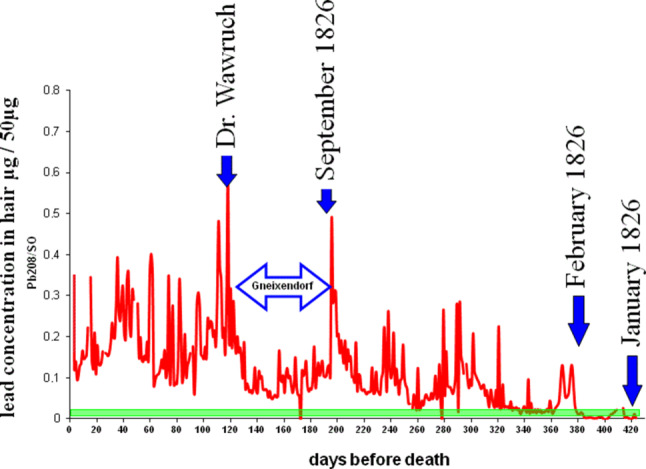
Time-dependent lead loading of the hair “Rollett 2” combined with the dates of relevant events during the last months of Beethoven’s life. The *green* base represents today’s standard value of lead burden in hair

The analytics of some of the hairs from the Rollett Museum’s lock confirmed congruent distribution pattern, as has already been found in the Guevara strands, wherein one recognizable peak in the levels of lead was detected at the 120th day before Beethoven’s passing, correlating with the date when Dr. Wawruch administered the anti-inflammatory drug (Fig. 4).

In the 80 days prior to Dr. Wawruch’s treatment, from September to December 5, 1826, when Beethoven was residing in Gneixendorf, no evidence of a relevant exogenous level of lead arise. During the period of 200–380 days before his death, there are some moderately strong lead peaks, while during the period of 380–420 days before Beethoven’s death the concentration of lead in his hair corresponds to today’s standard value.

In the period from January to February 1826, the lead concentration level is comparable with that found in people who have not been exposed today.

Thus, there were no indications of abnormal exposure to lead before February 1826.

## Discussion

As shown in the analysis of the ice mummy from Hauslabjoch (Ötzi), humans have been confronted with significant heavy metal contamination since they began to mine and smelt ore [11, 12]. For the attentive observer, the manifestation of related diseases could be witnessed throughout earlier periods of human history, including the use of heavy metals in household and cosmetic products, which would be called “wreckless” by today’s standards.

It is therefore not surprising that since antiquity, heavy metals were found in both poisons and in pharmaceuticals. Paracelsus expressed this double potency of heavy metals in his saying “ubi virus, ibi virtus” (where poison, there’s virtue). Paracelsus was the first author of the Occident to describe cases of occupational mercury poisoning, suffered by miners in Idrija (Idria), Slovenia, and had warned against the careless use of heavy metals as drugs; however, he did so by arguing for proper dosage. In the 18th and 19th century, much of the period’s treasury of drugs contained a variety of heavy metal salts, to which medical indications were well defined.

Thus, for example, we can see, the following description of the “Simple *Lead Patch”* taken from the *Handbook of Austrian Pharmacology* [13] by Meyer from 1838: *Mix lard with finely powdered lead oxide in a 2:1 ratio. Bring to boil through constant stirring and slowly adding water until resulting in a honey-like yellowish-white, sticky substance, firm nascent, but yields to the hand without becoming greasy and can stick easily to canvas. Applied to linen bands, this lead patch serves in softening the wound edges, as well as for preventing air from penetrating to ulcerous points and to ease and dispel inflammatory swelling*. Study the same book, one can also find the following recommendations: *The internal use of lead acetate with persistent fever should be used with great caution, if the latter is accompanied by strong mucous secretions or destruction of a purulent nature occurs. Such being the case for mucous and purulent, but not for tuberculous pneumonia*.

If one follows the common scientific practice of the time, the treatment given to Beethoven by Dr. Wawruchs would have most likely contained lead acetate. The fact that the action of poison was accompanied a healing quality—just as was mentioned in the medical literature of the time—one can also explain the improvement of the state of the lung infection in the days following this treatment. It is no surprise, however, that from 13 December 1826, drug-induced liver and kidney failure resulted followed by jaundice, diarrhea, vomiting, dropsy, and anuresis.

Without noticing the effects of a toxic lead poisoning Beethoven was suffering from, Dr. Siebert had most likely applied lead patches on the wound of the four paracenteses he had performed, which are reflected by the peaks measured in the core of the investigated hairs. From the point of view of that time, wound closure with lead patches was indicated and the astringent property of lead plasters could also be seen as indicated in the treatment of the erysipelas, as the materia medica express that *the external use of lead sugar in erysipelas as the result of external injuries is recommended*.

In the course of changing the bandages, Beethoven’s hands might have come in contact with the lead paste, through which additional particles had come in contact with his hair. This could explain the presence of lead particles on the surface of his hair. While the administration of the lead-containing drugs had been proven to resolve the pneumonia, it had simultaneously caused progredient liver failure, accelerated by the pre-existing cirrhosis.

Starting in February 1826, recurring abnormal lead measurements could be reconstructed, whereby it is important to mention that in accordance with his medical history, Beethoven had been suffering at that time (February 1826) from diarrhea, spasms in his hands and feet, dizziness, bitter taste in the mouth and back pain.

Beethoven, who in retrospect of his place of birth, had gained a fondness for sweet wine—a gastronomical preference, which had placed him at heightened risk. In those days it was not uncommon to decrease the vinegar content of the wine by addition of white lead to form a lead salt.

In this manner, the wine would also contain an additional “residual sugar” of “lead sugar” (lead acetate). The careless handling of winemakers with lead salts was denounced by the young physician Samuel Hahnemann, who had already in 1788 developed chemotoxical analysis methods for detecting lead in beverages such as wine treated in this manner [14]. There is a strong suspicion that during the period February 1826 to September 1826, Beethoven has consumed sweetened wine tainted in this manner, which resulted in the documented symptomatic ailments of February 1826. For the period of his stay in Gneixendorf there is no evidence of the consumption of such “tampered wine”.

Considering the numerous faces of handwritings of Ludwig van Beethoven which have been preserved in its autographs, one can observe a significant deterioration in his writing style occurring through the last months of his life due to lead intoxication.

From today’s perspective it can be determined that Beethoven’s deafness was certainly not the result of chronic lead poisoning. This is based on the results of tests conducted of the strand of hair from the Rollett Museum, in which there are no signs of exposure between the 380th and 420th day before his death, which do not correspond with the presence of a pre-existing chronic lead poisoning [4]. This would have resulted in moderate and consistent levels of lead in Beethoven’s hair because of the slow excretion of previously administered lead from the organism.

In view of the normal thickness of the regular three-layered skull fragments [15] which remain today, the cause of his deafness is most likely to be otosclerosis [16] or a vascular disease rather than Paget’s disease believed by some authors [17].

The liver cirrhosis, which had been going on prior to Beethoven’s lung infection and which had presented itself during Beethoven’s period in Gneixendorf with the loss of appetite and progressing abdominal swelling, could be best explained as a consequence of chronic hepatitis. Considering Beethoven did not have a permanent residence and frequented visiting taverns, the transmission of hepatitis A infection by contaminated well water is highly probable. This episode can also be placed within the context of his recorded medical history, in which he had suffered a “rheumatic attack” accompanied by jaundice in 1821, 6 years prior to his passing.

It is without doubt that Beethoven’s chronic alcohol consumption—estimated to be an entire bottle of wine per meal—had an unfavorable effect on his cirrhosis and the sclerosing pancreatitis. On the other hand, his digestive problems appear to have been caused by irritable bowel syndrome, which worsened psychosomatically with his increased loss of hearing and the impact it was having on his artistic work.

## Forensic conclusions

The question as to whether Beethoven’s death was a case of malpractice could only be delivered, from a forensic point of view, ex ante—if we take the state of the medical knowledge of that time into account. The choice to use lead-containing medicines or bandages at the time was contraindicated by Beethoven’s already manifested liver cirrhosis. Had Dr. Wawruch known of Beethoven’s medical history, prior ailments, symptoms and way of life—he should have (after careful physical examination of the liver consistency) not considered the use of lead-containing medicines. However, Dr. Wawruch was called upon on 5 December 1826 to treat a 4-day-old pneumonia, which had put Beethoven in imminent danger, not to mention the increased abdominal girth—the result of an incipient ascites—which had already began during his stay at Gneixendorf. In this situation, one must presume that the ability to examine abdominal organs was compromised and therefore it was considerably more difficult to examine the consistency of the liver. Thus, is it safe to assume that Dr. Wawruch did not undercover any evidence that would indicate careful or cautious use of lead-containing medication while treating Beethoven. From his perspective, Dr. Seibert—as a practicing surgeon— was expected to keep to the rule of art prevailing at the time. In this case, he would have had to rely on any advice that Dr. Wawruch (the internist) had given him while choosing an appropriate treatment.

From a forensic point of view, however, it must be noted that Beethoven’s therapeutic procedure would be contraindicated from today’s perspective. On the other hand, the ex ante perspective of that time had shown no indications that the two doctors did not follow the rules of art and, therefore it is safe to assume that they acted with due diligence.
